# Bio‐Compositional and Microstructural Changes in Rabbit Knee Collateral Ligaments Eight Weeks After Anterior Cruciate Ligament Transection

**DOI:** 10.1002/jor.70132

**Published:** 2026-01-04

**Authors:** Anahita Gheisari, Ville‐Pauli Karjalainen, Lassi Rieppo, Sami Kauppinen, Andrew Sawatsky, Rami K. Korhonen, Walter Herzog, Simo Saarakkalaa, Mikko A.J. Finnilä, Shuvashis Das Gupta

**Affiliations:** ^1^ Research Unit of Health Sciences and Technology, Faculty of Medicine University of Oulu Oulu Finland; ^2^ Polar Electro Oy Kempele Finland; ^3^ Faculty of Kinesiology, Human Performance Lab University of Calgary Calgary Alberta Canada; ^4^ Department of Applied Physics University of Eastern Finland Kuopio Finland; ^5^ Biocentre University of Oulu Oulu Finland; ^6^ Department of Diagnostic Radiology Oulu University Hospital Oulu Finland; ^7^ Department of Biomedical Engineering Lund University Lund Sweden

**Keywords:** ACL injury, fourier transform infrared spectroscopy, knee joint, ligament, quantitative polarized light microscopy

## Abstract

Injury to the anterior cruciate ligament (ACL) is common in young, active individuals. It has the potential to lead to post‐traumatic osteoarthritis. However, the effects of ACL injury on the bio‐composition and microstructure of the knee's collateral ligaments have been poorly explored. In this study, Fourier transform infrared (FTIR) imaging and quantitative polarized light microscopy (qPLM) were used to identify the respective changes in bio‐composition and collagen fiber arrangements of the knee's collateral ligaments. To mimic an ACL trauma, unilateral ACL transection surgery was performed on either the left or right knee of 6 mature New Zealand white rabbits. Lateral and medial collateral ligaments were harvested from the transected and contralateral knees 8 weeks after the ACL transection surgery. At the same time, collateral ligaments of 4 age‐matched, healthy rabbits were collected from the right and left knees. From acquired FTIR images, the relative collagen and proteoglycan contents of the collateral ligaments were estimated and compared between the transected, contralateral, and control knees. The results revealed lower collagen and higher proteoglycan content in ACL‐transected collateral ligaments compared to collateral ligaments of contralateral and control group knees. Additionally, qPLM revealed a more disorganized collagen fiber matrix, accompanied by increased crimp angles and longer crimp lengths following ACL transection. This study provides novel insight into the bio‐compositional and microstructural alterations of collateral ligaments following ACL injury, highlighting the importance of considering the structure‐function properties of collateral ligaments in treatment planning aimed at restoring normal knee joint function after ACL injury.

## 1 Introduction

Anterior cruciate ligament (ACL) injury is among the most common knee joint traumas, with an incidence rate ranging from 30 to 78 per 100,000 individuals annually, and a higher rate of injury is observed in female compared to male athletes [1, 2]. ACL injury increases the risk of developing osteoarthritis in the injured knee [3, 4, 5]. Studies have reported a 3%–34% risk of re‐injury in the ipsilateral knee, as well as a two‐ to threefold higher risk of ACL injury in the contralateral knee [6, 7, 8, 9, 10]. Collateral ligaments also contribute to knee stability by restraining varus and valgus rotation, and ACL injuries are often accompanied by collateral ligament damage [11, 12]. Previous studies on collateral ligaments after ACL transection have shown lower stiffness and failure stress in collateral ligaments of ACL transected knees compared to that of control knees [13, 14, 15, 16]; however, these studies have been mainly focused on medial collateral ligament (MCL) and changes in intact contralateral knee remains insufficiently neglected. Better understanding of changes in structure‐function of collateral ligaments post ACL injury could improve ligament graft design and treatment strategies.

The ACL, posterior cruciate ligament (PCL), lateral collateral ligament (LCL), and MCL are the primary ligaments of the knee, stabilizing the joint by limiting excessive movement. The primary constituents of knee ligaments include water, collagen type I and III, proteoglycan, elastin, and fibroblasts. The fibroblasts are surrounded by an extracellular matrix (ECM) and are in charge of the synthesis of the ECM [17].

Ligaments have a hierarchical structure, where triple helical collagen molecules join to make collagen microfibrils, which assemble into collagen fibrils. The collagen fibrils further compose collagen fibers that are the building blocks of collagen fascicles. Unloaded collagen fibers exhibit a wavy sinusoidal pattern called crimp, which is assumed to contribute to the non‐linear force‐elongation behavior of ligaments [18, 19]. Proteoglycans contain negatively charged glycosaminoglycans (GAG) that regulate the assembly of collagen fibrils, and influence collagen fibril separation and sliding [20, 21, 22, 23]. However, their contribution to the mechanical properties of ligaments is not yet fully understood.

The knee joint has a complex structure and injury to the ACL leads to alterations in the knee joint loading. Previous studies have also shown that ACL injury changes the joint loading leading to altered mechanical properties of the collateral ligaments [24, 25, 26, 27, 28, 29, 30, 31]. Altered mechanical loading has been shown to induce changes in the ECM of ligaments. Mechanical stress deprivation results in thinner collagen fibers, reduced collagen content, and decreased stiffness compared to controls, whereas stress resumption assists in restoring the microstructure and mechanical properties, although full recovery to control levels may take longer than the period of deprivation [32]. Cyclic tensile forces on periodontal ligaments resulted in a rearrangement of microfilaments and tissue remodelling [33, 34]. Therefore, analytic techniques that incorporate compositional and organizational changes in the ECM of ligaments can be useful in characterizing the altered mechanical properties caused by a change in the loading environment.

Fourier transform infrared (FTIR) spectroscopy provides insight into the macromolecular composition of biological tissue samples [35, 36, 37]. FTIR microspectroscopy integrates an FTIR spectrometer with an optical light microscope and provides chemical imaging of the specimens. FTIR imaging has previously been used as an effective technique to study the chemical distributions and compositional changes in connective tissues [38, 39, 40, 41, 42, 43]. Along with the bio‐composition, the structural arrangement and morphology of collagen fibers in collagenous tissue are closely linked to their function [44, 45, 46]. Quantitative polarized light microscopy (qPLM) has been shown to be an effective technique for visualizing fiber organization, and examining the morphological features of tendons and ligaments [44, 45].

Previous studies from our group revealed that collateral ligaments from ACL‐transected rabbit knees have lower stiffness and viscosity at 8 weeks compared to their contralateral counterparts and healthy controls, while the collateral ligaments of the contralateral group rabbits had greater stiffness and viscosity than the collateral ligaments of the control group rabbits [30, 31]. Additionally, our group applied Raman spectroscopy to effectively predict and differentiate between the mechanical properties of collateral ligaments from ACL‐transected and healthy control knees [47]. However, the changes in tissue constituents and microstructure responsible for these observations remain unknown.

This study aimed to provide a detailed analysis of the bio‐composition of knee collateral ligaments in a rabbit model at 8 weeks post‐ACL transection surgery using FTIR microspectroscopy. Furthermore, qPLM was used to assess collagen fiber orientation and crimp patterns. We hypothesized that alterations in mechanical properties are accompanied by compositional and microstructural changes, with ACL‐injured knees having a reduced collagen content and organization of the collagen fiber network compared to the collateral ligaments from the contralateral and control knees.

## 2 Materials and Methods

### 2.1 Sample Preparation

Strictly sterile unilateral ACL transection surgery was performed on the knees of six skeletally mature female New Zealand White rabbits (Oryctolagus cuniculus, 12 months old at the time of surgery, weighing 4.8 ± 0.1 kg) under general anaesthesia. Rabbits were kept on a heating pad and covered with a blanket to aid recovery until they regained full mobility. Rabbits were then returned to their cages (dimensions: 76 × 64 × 41 cm [3]) and allowed to move freely without knee immobilization. Animals were euthanized 8 weeks after ACL transection, and the MCLs (*n* = 12; 6 ACL, 6 contralateral) and LCLs (*n* = 11; 5 ACL, 6 contralateral) were harvested. An additional group of four rabbits, aged 14 months at the time of collateral ligament collection that was not subjected to surgery, served as unoperated controls (*n* = 16 collateral ligaments; 8 LCL, 8 MCL). The University of Calgary's Animal Care Committee (#AC11–0035) approved all experimental protocols, adhering to the guidelines of the Canadian Council on Animal Care. Ligament samples harvested from the knees were initially subjected to stress‐relaxation testing followed by dynamic load testing (Orozco2023, Gheisari2024), Subsequently, ligaments were allowed to relax, fixed in formalin, and processed for histology with extra care being taken in the embedding process to maintain ligaments straight to facilitate later sectioning.

**Figure 1:**
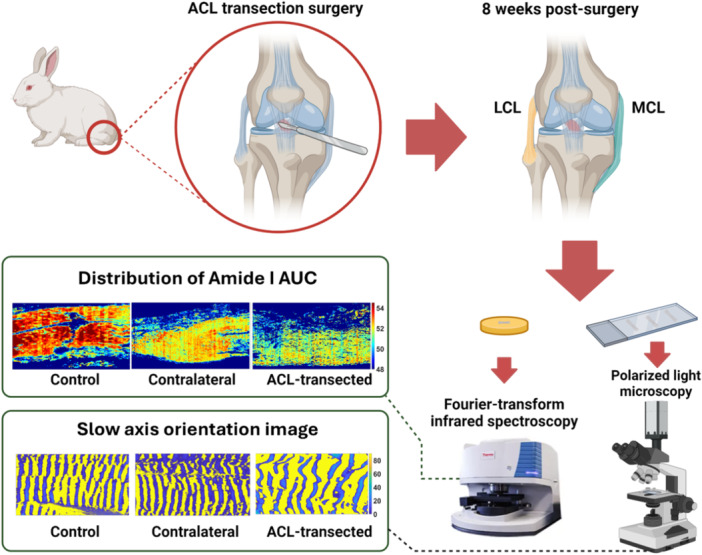
Overview of sample preparation and analytical techniques. An illustration of the sample preparation workflow and the techniques employed to assess compositional and structural changes in lateral collateral ligament (LCL) and medial collateral ligament (MCL) of the three groups: ACL‐transected (*n =* 11), contralateral (*n =* 12), and controls (*n =* 16). FTIR chemical images show the distribution of the integrated area under the curve (AUC) of the amide I peak, and qPLM images show the fiber orientations at the ‘slow axis’, respectively (Created with BioRender.com).

5‐μm‐thick mid‐substance sections were cut from the mid‐cross section of each sample along the longitudinal axis. After deparaffinization with Xylene, sections were mounted onto Zinc Selenide (ZnSe) substrates for FTIR and onto glass slides for qPLM, respectively (Figure 1).

### 2.2 FTIR Data Acquisition

For FTIR, this study utilized a Nicolet iN10 MX Infrared imaging microscope (Thermo Fisher Scientific, Waltham, MA, USA) equipped with a mercury–cadmium–telluride (MCT) line array detector cooled by liquid nitrogen. Background signal was collected from a clear area of the slide to subtract ZnSe signal from collected spectra. Spectral data were collected from a rectangular area of approximately 1500 × 1300 μm² in the middle of the tissue samples using a step size of 6.5 μm, resulting in ~49500 spectra per sample. Spectral data were collected over the range of 715–4000 cm⁻¹ with a spectral resolution of 4 cm⁻¹, capturing the essential vibrational modes of the ligament tissue. To enhance the signal‐to‐noise ratio, 64 scans were averaged at each pixel.

### 2.3 Spectral Pre‐Processing

**Figure 2:**
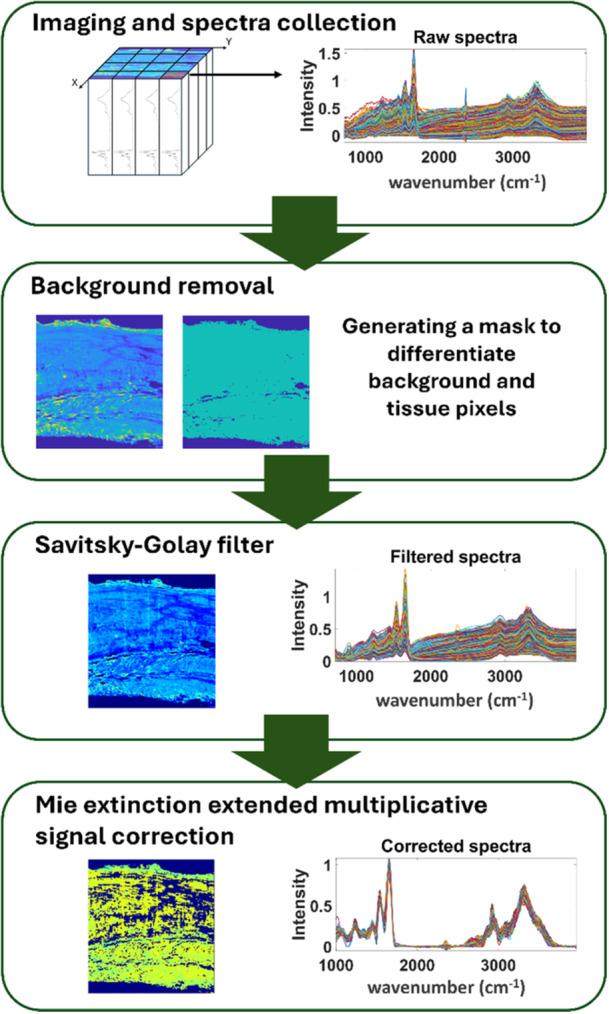
A diagram showing the steps of spectral pre‐processing. The selection of the root mean square error (RMSE) as part of the Mie extinction multiplicative signal correction (ME‐EMSC) algorithm led to a decrease in the number of tissue pixels.

Initially, the FTIR spectra from the tissue were distinguished from the background by using the mean spectral intensity values, generating a mask for tissue pixels from the FTIR images. Subsequently, the FTIR spectra were denoised using a second‐order Savitzky‐Golay filter with a window length of 21 in MATLAB R2023a. To account for Mie scattering effects, FTIR spectra were fed to the Mie extinction extended multiplicative signal correction (ME‐EMSC) algorithm developed by Solheim et al. [48]. The explained variance in the ME‐EMSC algorithm was set to 99.99%, allowing a maximum of 45 iterations, with weight functions and physical parameters at default settings. The root mean square error was kept at 0.05 as the stop criterion of the algorithm (Figure 2).

### 2.4 Univariate Analysis

The FTIR spectra of rabbit collateral ligaments were analyzed within the region of 1000–4000 cm⁻¹. The spectral peaks of amide I (1658 cm⁻¹) and amide II (1546 cm⁻¹) are characteristic of collagenous tissues and indicative of collagen content (Table 1). The peak at 1450 cm⁻¹ is another characteristic band associated with collagen. Moreover, the peaks at 1334 cm⁻¹ and 1234 cm⁻¹ are attributed to collagen side chain vibrations (Table 1). The spectral region of the amide B peak (3077 cm^−1^) is also reported to be related to collagen. The spectral region of 1000–1184 cm⁻¹ contains peaks at 1031, 1060, 1072, 1082, 1134, and 1161 cm⁻¹ that were reported to be associated with the carbohydrate residue of proteoglycans (Table 1).

**Table 1:** Assignment of FTIR spectral peaks corresponding to tissue compounds. The wavenumbers are based on literature, with the wavenumber in parentheses representing the value observed in this study.

Wavenumber (cm^−1^)	Assignment	Compound	Reference
1660 (1658)	C═O	Amide I in collagen	[35, 49, 50, 51]
1554 (1546)	C─N N─H	Amide II in collagen	[35, 51]
1446 (1450)	CH_2_	Asymmetric bending vibration in collagen	[35]
1338 (1334)	CH_2_	Side chain vibration of collagen	[35]
1236 (1234)	C─N N─H CH_2_	Collagen amide III vibration from C─N stretching, N─H bending vibrations and wagging vibrations of CH_2_ groups in the glycine backbone and proline side chains.	[35]
1161	C─O	Stretching vibration of the carbohydrate residues	[52]
1134	C─O─S	Asymmetric stretching of GAGs	[53]
1082	C─O	Stretching vibration of the carbohydrate residues in collagen and PGs	[49]
1072	C─O─C C─OH C─C	Sugar ring of GAGs	[35, 53]
1060	C─O SO_3_^−^	C─O stretching vibration of the carbohydrate residues in collagen and PGs/SO3− symmetric stretching vibration of sulfated GAGs	[49, 50]
1031	C─O	Stretching vibration of the carbohydrate residues in collagen and PGs	[49, 50]
3080 (3077)	N─H	Amide B in collagen, N─H stretching	[54, 55]

**Figure 3:**
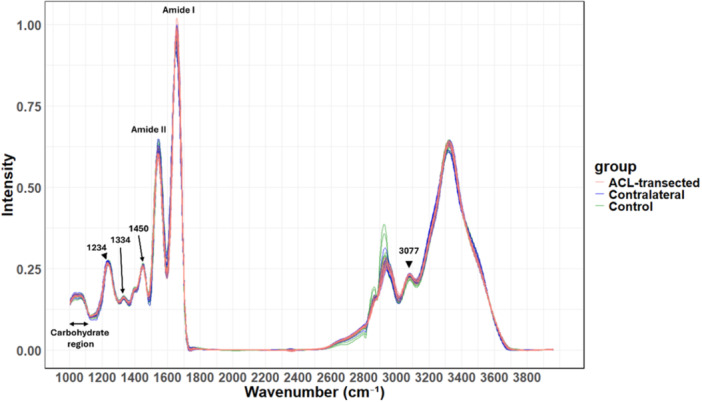
Mean spectra of collateral ligament samples from the three groups (ACL‐transected, contralateral, and control). The peaks used in the univariate analysis are labeled on the spectra.

The integrated area under the curve (AUC) for amide I, amide II, 1450 cm⁻¹, 1334 cm⁻¹, 1234 cm⁻¹, amide B, and 2919 cm⁻¹ peaks, as well as the carbohydrate region (1000–1184 cm⁻¹), was calculated for all spectra from the tissue mask. Local baseline correction was applied to each spectral region before calculating the AUC and generating pseudo‐colored FTIR chemical images. Figure 3 presents the mean spectra of samples in each group.

### 2.5 qPLM Imaging

The qPLM imaging was performed using an Abrio qPLM system (CRi Inc., Woburn, MA, USA) mounted on a Nikon Diaphot TMD light microscope (Nikon Inc., Shinagawa, Tokyo, Japan). The Abrio system includes three main components: a green bandpass filter, a circular polarizer, and an analyzer. Two 5‐µm‐thick ligament sections for each sample were imaged in the same orientation using a 10X objective, producing a pixel size of 1 × 1 μm².

### 2.6 Collagen Fiber Orientation and Crimp Analysis

**Figure 4:**
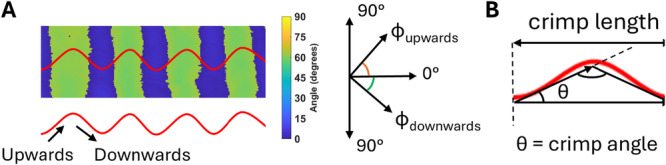
The figure provides a schematic illustration of the evaluation of crimp characteristics. (A) Unstained section of an MCL from the control group, with the red line representing a schematic plot of crimp pattern. (B) Definition of crimp length and crimp angle (θ).

The pixel values of qPLM images correspond with the collagen fiber orientation in which 0ᵒ indicates a fiber parallel to and 90ᵒ perpendicular to the longitudinal axis of the ligaments. For orientation analyses, the samples were normalized along the longitudinal axis of the ligament so that fiber angles ranged from 0° to 90°. The crimp angle and length were also measured from the collected qPLM images according to work by Spiesz et al. [44] with a custom‐made MATLAB script. Images with out‐of‐plane crimp were excluded from the analysis. ‘Slow axis’ (the direction with the highest refractive index) angle images were used to measure the collagen crimp angle and length. Sections with in‐plane crimp patterns were identified for each sample to make sure the crimp pattern was aligned with the cutting direction. Subsequently, five regions of interest (ROIs) per section of each specimen were selected and averaged for crimp angle and length measurements. The angle of the fiber ascending toward the peak in the single wavy pattern was determined as φ_upwards_, while the angle descending toward the peak was denoted as φ_downwards_ (Figure 4A,B). These measurements were then used to calculate the crimp angle as follows:

$$\theta =\;\frac{1}{2}\left(\varphi_{\text{upwards}}+\varphi_{\text{downwards}}\right)$$

The distance between two adjacent valleys was defined as crimp length (Figure 4B).

## 3 Statistical Analysis

For statistical analysis, univariate FTIR and qPLM parameters (fiber angle, crimp angle, and crimp length) were averaged for each sample. To compare the bio‐composition between experimental groups, separate linear mixed effects models (LMEs) were fitted for each FTIR parameter (roughly normally distributed) as the response variable. Ligament type (LCL and MCL) and experimental group were specified as fixed effects, with random intercepts for ligaments and knees within each animal. Model fitting was performed using the “lme4” package [56] in R version 4.2.2 (R Core Team, 2022). Post‐hoc pairwise comparisons across the experimental groups were subsequently conducted using the “emmeans” package [57] in R 4.2.2.

For the qPLM results, a separate LME was fitted, and estimated means were calculated with SPSS 29 software (SPSS Inc., IBM Company, Armonk, NY, USA). We used the natural logarithm transform for each qPLM parameter to achieve a normal distribution and fitted the LME separately to the qPLM parameters as response variables. Ligament type and experimental group with their interaction were considered as fixed effects, and animal as a random effect. Residual diagnostics confirmed an adequate model fit.

## 4 Results

### 4.1 FTIR Analysis of Collateral Ligaments

**Figure 5:**
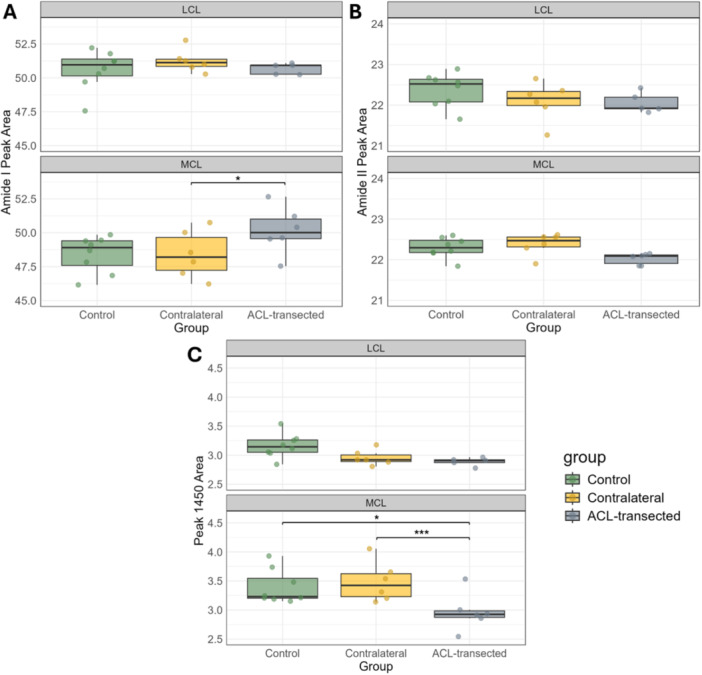
Boxplots depict the median, 25 and 75 percent quartiles, and extreme values of the amide I peak AUC (A), amide II peak AUC (B), and peak 1450 cm⁻¹ AUC (C) for the LCL and MCL in control, contralateral, and ACL‐transected groups. Each dot represents the mean AUC value of a sample. MCL stands for medial collateral ligament, and LCL stands for lateral collateral ligament. Significant differences are marked with *(*p <* 0.05) and ***(*p <* 0.001).

The MCLs of contralateral knees had lower (*p *< 0.05) amide I content (48.41 ± 1.74) than those of ACL‐transected group knees (50.16 ± 1.73) (Figure 5A). For the amide II band, no significant differences were observed among the three experimental groups (Figure 5B). However, the MCLs of ACL‐transected group knees had a lower (*p* < 0.05) mean AUC value of 1450 cm⁻¹ peak (2.96 ± 0.32) compared to contralateral group knees (3.48 ± 0.34) and control knees (3.39 ± 0.29) (Figure 5C).

**Figure 6:**
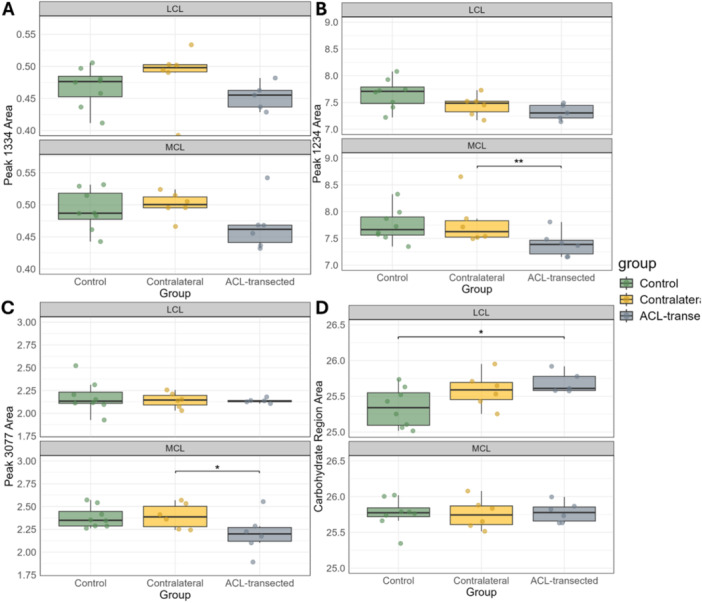
Boxplots illustrate the median, 25 and 75 percent quartiles, and extreme values of the peak 1334 cm⁻¹ AUC (A), peak 1234 cm⁻¹ AUC (B), amide B (3077 cm⁻¹) AUC (C), and carbohydrate region AUC (D) for the LCL and MCL in control, contralateral, and ACL‐transected groups. Each dot represents the mean AUC value of a sample. MCL stands for medial collateral ligament, and LCL stands for lateral collateral ligament. Significant differences are marked with *(*p <* 0.05) and **(*p <* 0.01).

The mean AUC values for other collagen‐related bands with peaks at 1334 cm⁻¹, 1234 cm⁻¹ (side‐chain vibrations), and 3077 cm⁻¹ (amide B) showed similar group‐wise changes with MCLs (Figure 4). The AUC values of the band at 1334 cm⁻¹ tended to be higher for MCLs in contralateral knees (0.50 ± 0.02) compared to control (0.49 ± 0.03) and ACL‐transected knees (0.47 ± 0.04) (Figure 6A). Similarly, the MCLs of contralateral knees showed higher (*p* < 0.01) AUC values at the 1234 cm⁻¹ band (7.8 ± 0.44) compared to both control (7.74 ± 0.31) and ACL‐transected knees (7.40 ± 0.24) (Figure 6B). Furthermore, the MCLs of contralateral knees had higher (*p* < 0.01) AUC values of the 3077 cm⁻¹ band (2.40 ± 0.14) than that of control (2.38 ± 0.12) and ACL‐transected knees (2.21 ± 0.22) (Figure 6C). Finally, the AUC values of the carbohydrate region were higher (*p* < 0.01) for the LCLs of ACL‐transected knees (25.69 ± 0.15) compared to contralateral (25.59 ± 0.24) and control group knees (25.34 ± 0.27) (Figure 6D).

### 4.2 qPLM Analysis of Collateral Ligaments

**Figure 7:**
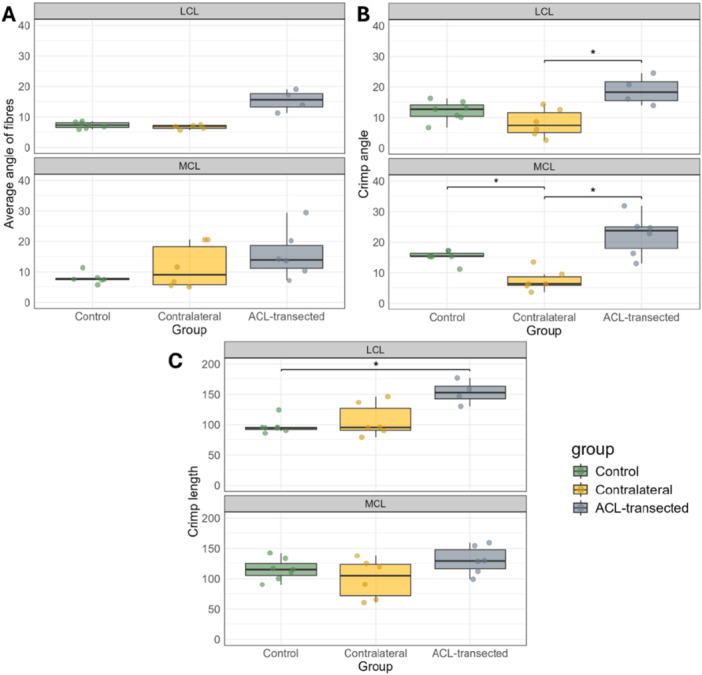
The boxplots display the median, 25 and 75 percent quartiles, and extreme values for the average angle of fibers (A), crimp angle (B), and crimp length (C) of the LCL and MCL across control, contralateral, and ACL‐transected groups. Each dot represents the mean values of a sample for the average angle of fibers, crimp angle and length. MCL stands for medial collateral ligament, and LCL stands for lateral collateral ligament. Significant differences are marked with *(*p <* 0.05).

The average collagen fiber angle in the MCLs of the ACL‐transected knees (15.8° ± 0.8) tended to be larger than that of the contralateral (11.6° ± 7.3) and the control group knees (7.9° ± 1.9) (*p* = 0.602 and *p* = 0.137, respectively) (Figure 7A). A similar pattern was observed in the LCLs, but as for the MCL there were no significant differences between ACL and CL groups(*p* = 0.134) or ACL and control groups (*p* = 0.063). The crimp angle of the LCLs in ACL‐transected knees (18.8ᵒ ± 4.8) was greater (*p* < 0.05) than that of the contralateral (8.1ᵒ ± 4.6) (Figure 7B). The crimp angle of the MCLs in ACL‐transected knees (22.2ᵒ ± 6.8) was greater (*p* < 0.05) than that of the contralateral (7.4ᵒ ± 3.5) and control knee groups (15.2ᵒ ± 2.0). Notably, the contralateral group exhibited the lowest mean crimp angle compared to the ACL‐transected and control group knees for both LCLs and MCLs. Finally, the ACL‐transected LCLs exhibited a greater mean crimp length (153.1 µm ± 19.7) compared to the contralateral (107.2 µm ± 27.5) and greater (*p* < *0.05*) than that of control knees (97.3 µm ± 12.4) (Figure 7C).

## 5 Discussion

The FTIR imaging and qPLM revealed bio‐compositional and structural differences across the ACL‐transected, contralateral, and control knees, for both LCLs and MCLs. Understanding the bio‐compositional changes in these ligaments is crucial for elucidating the effects of ACL injury on the knee collateral ligaments, which may enhance our understanding of the ligament structure‐function relation. In agreement with the original hypothesis, the results of this study suggest that ACL injury leads to changes in the collagen and proteoglycan content of the collateral ligaments. The FTIR univariate analyses of collagen were supported by qPLM results, which depicted changes in fiber alignment and structural characteristics. It is plausible that these changes occur in response to the altered knee mechanical environment following ACL injury.

A lower collagen content was observed in the collateral ligaments of the ACL‐transected group knees compared to the control and contralateral group knees. The collagen is one of the main factors determining the mechanical properties and structural integrity of collagen fibrils in connective tissues [58, 59, 60, 61]. Therefore, the lower AUC levels of collagen in collateral ligaments of ACL‐transected knees can be due to disruptions in collagen synthesis and cross‐linking, indicating diminished mechanical properties. Previous research on the mechanical properties of the same set of collateral ligaments showed a lower stiffness and viscosity in the ACL‐transected group knees compared to the contralateral and control group knees [30, 31]. The results from this study support the notion that changes in mechanical properties are accompanied by changes in collagen content. A previous study on repaired MCL of ACL‐reconstructed knees in New Zealand white rabbits depicted lower collagen content compared to sham‐operated control knees at 6 weeks after surgery, but the collagen content recovered to near‐normal control group values at 12 weeks post‐intervention [62]. In another study, it was found that the collagen density of the healing MCL was lower than in healthy controls at 3 and 6 weeks following a mid‐substance injury; a comparable collagen density was reached 14 weeks after MCL injury [63]. As ACL injury can lead to knee laxity, the lower collagen content in the collateral ligaments of the injured knee, as found in our studies, can contribute to further knee instability. The combination of ACL loss and changes to the collateral ligaments may impair joint function during daily activities and increase susceptibility to future knee ligament injuries.

The qPLM results indicated that the microstructure of the collagen fibers in the ACL‐transected group knees was altered. LCLs and MCLs in the ACL‐transected group had a greater range of collagen fiber angles, likely due to a greater number of fibers oriented nonparallel to the longitudinal axis of the ligaments (Figure 5A). Collagen organization in connective tissues is a key factor affecting the mechanical and functional properties [64, 65, 66]. Healing MCLs with less organized collagen fiber alignment demonstrated lower tensile strength and smaller elastic moduli compared to healthy MCLs [63]. Knee ligaments experience tensile, shear, and compressive loading, thus collagen fibers are arranged to resist different loadings during various knee movements [67]. However, since tensile forces are the main loading resisted by ligaments, most fibers are aligned longitudinally along the long axis of the ligament [68]. The smaller number of collagen fibers aligned along the longitudinal axis of ligaments in ACL‐transected groups suggests that disorganized collagen structure, which, together with reduced collagen content, may contribute to a reduction in stiffness.

The fiber crimp angle and length of collateral ligaments of ACL‐transected knees were also greater than that of contralateral and control group knees. Crimp pattern in ligaments can provide extra resistance to the initial tensile loading by straightening, thereby providing the “toe‐region” of the stress‐strain curve. Longer crimp patterns in LCLs and MCLs of ACL‐transected knees may extend the toe region, delay stiffening, and reduce joint stability [69, 70]. Furthermore, greater crimp lengths have been associated with longer relaxation times during stress‐relaxation test [45]. Our previous mechanical testing on the same set of collateral ligaments also revealed that the LCLs and MCLs in the ACL‐transected group had longer relaxation times compared to the contralateral and control group collateral ligaments [30, 31]. Given the complex hierarchical structure of collagen in ligaments, factors such as collagen fibril diameter, length, and interactions between fibrils are also thought to influence the mechanical properties in addition to the collagen content and fiber alignment [26]. Future studies may want to focus on the contributions of factors other than those quantified here to reveal the structure‐function relationship of healing ligaments.

Our results showed a greater AUC value in the carbohydrate region of the LCLs from the ACL‐transected group knees compared to the control and contralateral group knees. Previous studies showed that the AUC values of the carbohydrate region directly correlate with the proteoglycan content of the connective tissue [38, 40]. Proteoglycans consist of a protein that is attached covalently to one or more carbohydrate chains [21, 71, 72]. Proteoglycans bind water, and previous AFM studies have shown that increased proteoglycan content can raise water content and reduce stiffness [23, 73, 74]. Higher proteoglycan and water content, together with lower collagen, may have contributed to reduced stiffness in collateral ligaments post‐ACL injury. The proteoglycans are also responsible for collagen inter‐ and intra‐fibrillar cross‐linking [75]. Studies exploring the role of proteoglycans in the mechanical and viscoelastic properties of ligaments (and tendons) have not yielded conclusive results. Initially, it was hypothesized that proteoglycans facilitate load transfer under tensile strain by binding adjacent collagen fibrils, thereby contributing to the tensile mechanical properties of collagenous tissues [76, 77]. On the other hand, more recent experimental studies on human MCLs and rat tail tendons showed that enzymatic proteoglycan depletion does not significantly affect the elastic properties of tissues [78, 79, 80]. The findings of the present study align with these latter observations. Despite higher proteoglycan content in ACL‐transected LCLs compared to LCLs from contralateral and control group knees, the ACL‐transected LCLs of the same animals had reduced stiffness in comparison to the LCLs from contralateral and control group knees [30]. However, the interaction between proteoglycans and collagen fibrils is hypothesized to support sliding between fibrils. An increase in proteoglycan content may prolong the time required for collagen fibrils to rearrange after loading, potentially resulting in longer relaxation times. Nevertheless, Ristaniemi et al. found no direct relationship between stress‐relaxation time and proteoglycan content [45]. Instead, the authors suggested that the slowed relaxation is likely caused by the densely packed collagen fibrils. Further research is required to reveal the mechanical role of proteoglycans in ligaments and tendons and the factors driving proteoglycan production.

While this study provides valuable insights into the changes in ligaments in the presence of joint perturbation, it has limitations that should be considered when interpreting the results. The small sample size often led to trends in the data that did not reach statistical significance. However, previous studies have shown that a smaller sample size is sufficient to illustrate differences between groups in similar contexts [30, 31, 47, 81]. The ligament samples were dehydrated and deparaffinized prior to FTIR imaging, processes that can affect the biomolecular composition, thus altering the spectra of tissues [82]. Since the same procedures were applied to all samples, it may be assumed that any effect caused by these procedures would be similar for all samples independent of the knee joint condition. In this study, only a ROI (around 1500 × 1300 μm²) from the mid‐substance of the collateral ligaments was used for the FTIR univariate analysis, which excludes the compositional differences within the length of ligaments, especially towards the insertion sites [42]. FTIR identifies functional groups through bond vibrations and provides information on molecular composition, but not directly on molecule size. Ligaments contain multiple macromolecules, and the ME‐EMSC algorithm requires the physical parameters of all macromolecules. Those parameters were set to default settings after a subjective evaluation. In addition, Matrigel spectra were used as reference spectra for the ME‐EMSC algorithm. Adjusting the settings or using a different reference spectrum may lead to variations in outcomes. This study considered a single time point (8 weeks) after ACL transection surgery. Previous research has demonstrated that collagen levels in healing collateral ligaments initially exhibit lower collagen content compared to healthy controls. However, by 12 weeks following ligament transection, the gap between experimental and control groups narrows, with no significant differences in collagen content reported [62, 63]. To gain a better understanding of the compositional and microstructural changes in collateral ligaments following ACL injury, it is recommended to include multiple time points for evaluation in future studies. Furthermore, ligaments are primarily composed of collagen type I, with less than 5% consisting of other collagen types [83]. Studies of connective tissue injury have shown that collagen type III increases with random organized network in the early phases but is gradually replaced by collagen type I over time [84, 85, 86]. In the present study, total collagen content in collateral ligaments decreased, which can be mainly attributed to decrease in collagen type I. Future studies are recommended to examine the collateral ligaments after ACL injury using immunochemistry methods to investigate changes in collagen types. In contrast to light microscopy and immunohistochemistry, FTIR microspectroscopy is not yet widely available, as it requires specialized equipment and expertise for data collection and analysis. In this study, we demonstrated the capability of FTIR in transmission mode to detect compositional changes in collateral ligaments following ACL transection. Emerging fiber‐optic instruments can enable in vivo FTIR measurements in ATR mode to further investigate the composition of ligaments and other tissues. Overall, FTIR spectroscopy is a fast and non‐destructive technique with the potential to be used as a complementary diagnostic tool in musculoskeletal injuries.

## 6 Conclusion

ACL transection in rabbits resulted in bio‐compositional and micro‐structural changes in the collateral ligaments of the operated knee. The bio‐compositional and collagen fiber alterations observed are consistent with those seen during ligament healing. Moreover, these changes could play a direct role in the structure‐function relationship of the ligament. Overall, this study offers important insights into the changes in collateral ligaments following ACL injury. Our findings highlight the need to consider the health of collateral ligaments in ACL‐injury treatment planning and emphasize a holistic approach to improve the rehabilitation and treatment strategies for ACL injuries to restore normal knee function.

## Author Contributions

**Anahita Gheisari:** writing – original draft, writing – review and editing, visualization, software, investigation, formal analysis, funding acquisition. **Ville‐Pauli Karjalainen:** writing – review and editing, formal analysis. **Lassi Rieppo:** writing – review and editing, software, methodology, funding acquisition. **Sami Kauppinen:** writing – review and editing, software. **Andrew Sawatsky:** writing – review and editing, investigation. **Rami K. Korhonen:** writing – review and editing, supervision, resources, project administration, funding acquisition, conceptualization. **Walter Herzog:** writing – review and editing, supervision, resources, project administration, funding acquisition, conceptualization. **Simo Saarakkala:** writing – review and editing, supervision, project administration, funding acquisition, conceptualization. **Mikko A.J. Finnilä:** writing – review and editing, supervision, resources, methodology, funding acquisition, conceptualization, project administration. **Shuvashis Das Gupta:** writing – review and editing, supervision, methodology, conceptualization.

## Conflicts of Interest

The authors declare no conflicts of interest.
